# Electronic, Optical, and Lattice Dynamical Properties of Tetracalcium Trialuminate (Ca_4_Al_6_O_13_)

**DOI:** 10.3390/ma11030449

**Published:** 2018-03-19

**Authors:** Huayue Mei, Yuhan Zhong, Peida Wang, Zhenyuan Jia, Chunmei Li, Nanpu Cheng

**Affiliations:** Faculty of Materials and Energy, Southwest University, 2^#^Tiansheng Road, Beibei, Chongqing 400715, China; mhy123@email.swu.edu.cn (H.M.); zyhvane@email.swu.edu.cn (Y.Z.); pokm9000@email.swu.edu.cn (P.W.); jiazhenyuan@email.swu.edu.cn (Z.J.); lcm1998@swu.edu.cn (C.L.)

**Keywords:** Ca_4_Al_6_O_13_, electronic structure, mechanical property, optical property

## Abstract

The electronic, optical, and lattice dynamical properties of tetracalcium trialuminate (Ca_4_Al_6_O_13_) with a special sodalite cage structure were calculated based on the density functional theory. Theoretical results show that Ca_4_Al_6_O_13_ is ductile and weakly anisotropic. The calculated Young’s modulus and Poisson ratio are 34.18 GPa and 0.32, respectively. Ca_4_Al_6_O_13_ is an indirect-gap semiconductor with a band gap of 5.41 eV. The top of the valence band derives from O 2*p* states, and the bottom of conduction band consists of Ca 3*d* states. Transitions from O 2*p*, 2*s* states to empty Ca 4*s*, 3*d* and Al 3*s*, 3*p* states constitute the major peaks of the imaginary part of the dielectric function. Ca_4_Al_6_O_13_ is a good UV absorber for photoelectric devices due to the high absorption coefficient and low reflectivity. The lattice vibration analysis reveals that O atoms contribute to the high-frequency portions of the phonon spectra, while Ca and Al atoms make important contributions to the middle- and low-frequency portions. At the center of the first Brillouin zone, lattice vibrations include the Raman active modes (E, A_1_), infrared active mode (T_2_), and silentmodes (T_1_, A_2_). Typical atomic displacement patterns were also investigated to understand the vibration modes more intuitively.

## 1. Introduction

Calcium aluminates are a series of inorganic compounds obtained by sintering calcium oxide and alumina at high temperatures. They have a wide variety of allotropes and different geometric structures, and are mainly used for refractory materials, calcium aluminate cements, optoelectronic devices, etc. [1,2]. Among them, monocalcium aluminate (CaAl_2_O_4_) is mainly used for hydraulic cements, refractory castables, optical devices, and structural materials [3]. Monocalcium dialuminate (CaAl_4_O_7_) is widely used in high-temperature refractory materials. It is important to note that CaAl_4_O_7_ doped with rare earth metal ions can be applied to luminescent materials such as light emitting diode (LED) lamps [4,5,6]. Both nocalcium aluminate and monocalcium dialuminate are monoclinic. Monocalcium hexa-aluminate (CaAl_12_O_19_) belongs to the hexagonal crystal system, and is mainly used in high-temperature refractory materials [7,8]. Tricalcium aluminate (Ca_3_Al_2_O_6_), having a cubic structure, is the main component of cement [9]; dodecacalcium hepta-aluminate (Ca_12_Al_14_O_33_) belongs to the cubic crystal system and is a high-temperature oxide ion conductor [10,11]. Moreover, there are some other calcium aluminate phase structures such as dicalcium aluminate (Ca_2_Al_2_O_5_) [12], pentacalcium trialuminate (Ca_5_Al_6_O_14_) [13], and tetracalcium trialuminate (Ca_4_Al_6_O_13_). Ca_4_Al_6_O_13_ has two allotropes that can be formed by dehydrating 4CaO·3Al_2_O_3_·3H_2_O in experiments. The first allotrope of Ca_4_Al_6_O_13_, synthesized at 1250 °C and 2.5 Gpa by Kahlenberg, has the same structure as perovskite and belongs to the orthorhombic system [14]. The second allotrope determined by Lars Peters et al. [15] belongs to the cubic system with a special sodalite cage structure, and also appears in some chemical reactions [16]. Although the cubic crystal structure of Ca_4_Al_6_O_13_ (CAO) has been discovered, there is a lack of detailed investigations on its physiochemical properties and potential applications. In this work, the electronic, mechanical, optical, and lattice dynamical properties of cubic CAO are calculated by using the first-principles method based on density functional theory. The motivation of the current work is to provide both an in-depth study of the physiochemical properties and a theoretical basis for further applications of CAO in many situations, such as high alumina cements, photoelectric devices, and so on.

## 2. Crystal Structure and Computational Details

### 2.1. Crystal Structure

**Figure 1:**
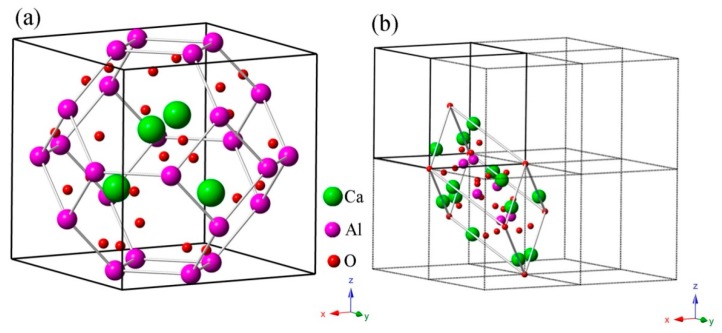
Crystal structure of CAO: (**a**) the unit cell (lattice parameters a =b= c = 8.8214 °) with a sodalite cage (β-cage) structure and (**b**) the primitive cell (primitive vectors $a_{1}\;=a_{2}=\;a_{3}\;=\;7.64\;{}^{\circ}$) in a 2 × 2 × 2 super cell.

CAO belongs to the cubic crystal system with the space group *I*-43m (No.217), as shown in Figure 1a. According to the experimental report [15], the unit cell parameters are a =b= c = 8.8214 Å and α =β=γ= 90 °. Ca atoms are located at 8c (0.1554, 0.1554, 0.1554), Al atoms are at 12d (0.25, 0.5, 0), and O atoms are at 24g (0.3583, 0.3583, −0.0831) and 2a (0, 0, 0). It is interesting that cubic CAO, which is similar to Zn_4_B_6_O_13_, has a special sodalite cage (*β*-cage) structure [17]. There are four [CaO_4_] tetrahedrons around the O atom in the center of the unit cell that form a radial [Ca_4_O_13_] cluster. Each [Al_24_O_48_] sodalite cage is made up of 24 [AlO_4_] tetrahedra corner-shared by O atoms. The [Al_24_O_48_] sodalite cages are connected by the inside [Ca_4_O_13_] clusters through the relatively strong Al–O covalent bonds. Figure 1b shows the primitive cell of CAO in a 2 × 2 × 2 super cell.

### 2.2. Computational Details

The current work was performed using the Cambridge serial total energy package (CASTEP) computational code, a kind of plane wave pseudopotentials first-principles quantum mechanics code based on the density functional theory [18]. In the calculations of geometrical optimization and mechanical properties of CAO, the generalized gradient approximation of Perdew–Burke–Ernzerhof (GGA–PBE) was employed for the exchange–correlation effects. However, compared with the experimental results, the GGA–PBE function often underestimates the band gaps of solids because the exchange correlation energy is discontinuous at this time [19]. The shortcoming of underestimate of band gaps can be improved by using different hybrid functionals [20]. Therefore, the PBE0 hybrid function was used to calculate the electronic structures and optical properties of CAO. In all calculations, the norm-conserving pseudopotentials with an energy cut-off of 880 eV for plane–wave basis sets were chosen to describe the interaction between valence electrons and ion core [21]. The 7 × 7 × 7 Monkhorst–Pack grids with an actual spacing ~0.0237 1/Å were adopted for geometry optimization and physiochemical properties. The convergence threshold of the total energy satisfies the following conditions: the residual stress was less than 0.01 eV, the stress tensor was below 0.02 GPa, and the displacement deviation was smaller than 5 × 10^−4^ Å. The basic electronic configurations in the calculations were Ca3*s*^2^3*p*^6^4*s*^2^, Al3*s*^2^3*p*^1^and O2*s*^2^2*p*^4^, respectively.

## 3. Results and Discussion

### 3.1. Structural Parameters

After geometrical optimization by the generalized gradient approximation (GGA), the calculated crystal lattice constant of CAO is 8.59 Å close to the experimental value of 8.82 Å [15], and the relative error is 0.4%. At this time, Ca atoms are located at (0.1597, 0.1597, 0.1597), Al atoms are at (0.25, 0.5, 0), and O atoms are at (0.3551, 0.3551, 0.0947) and (0, 0, 0). It can be seen that the atomic site occupations have changed slightly compared with the initial atomic positions mentioned above. Consequently, both the current crystal model and the computational method are reliable.

### 3.2. Mechanical Properties

The cubic crystal CAO has only three independent elastic constants, $C_{11}$, $C_{12}$ and $C_{44}$, in the 6 × 6 elastic constant matrix [21]. The corresponding mechanical stability criteria require [22]:

(1) $$C_{11}>0,C_{44}>0,C_{11}>\left|C_{12}\right|,\;\left(C_{11}\;+\;2C_{12}\right)>0\;$$

The calculated $C_{11}$, $C_{12}$ and $C_{44}$ values of CAO are respectively 131.15, 74.19 and 41.43 GPa, and they all meet themechanical stability criteria in Equation (1), indicating that CAO is mechanically stable. Other mechanical properties of CAO, such as the bulk modulus (B), shear modulus (G), and elastic modulus (E), can be obtained using the Voigt–Ruess–Hill approximation [23] based on the calculated elastic constants. In the Voigt–Ruess–Hill approximation, the Voigt and Ruess theories correspond to the upper and lower limits, respectively, and the Hill theory is the mean of the former two theories. The result ofthe Hill theory is closest to the experimental data. 

The Voigt and Ruess bulk modulus and shear modulus ($B_{V}$, $G_{V}$, $B_{R}$ and $G_{R}$) of cubic crystals can be calculated directly from the elastic constants through the following equations [22]:

(2) $$B_{V}=\;B_{R}\;=\;\left(C_{11}\;+\;2C_{12}\right)/3$$

(3) $$G_{V}=\;\left(C_{11}-C_{12}+\;3C_{44}\right)/5$$

(4) $$G_{R}=\;5\left(C_{11}-C_{12}\right)\;C_{44}/\left[4C_{44}\;+\;3\left(C_{11}-C_{12}\right)\right]$$

Finally, more accurate bulk modulus (B) and shear modulus (G) are obtained based on Hill’s average [23]:

(5) $$B_{H}=\frac{B_{V}+B_{R}}{2}$$

(6) $$G_{H}=\frac{G_{V}+B_{R}}{2}$$

For a polycrystalline system, the Young’s modulus (E) and Poisson’s ratio (v) can be calculated based on the bulk modulus and shear modulus [23]:

(7) $$E=\frac{9B_{H}G_{H}}{3B_{H}+G_{H}}$$

(8) $$\nu =\frac{3B_{H}-2G_{H}}{2\left(3B_{H}+G_{H}\right)}$$

The bulk modulus, shear modulus, Young’s modulus, and Poisson’s ratio of CAO calculated from Equations (1)–(8) are listed in Table 1. Pugh [23] proposed a principle called the $B_{H}/G_{H}$ ratio to judge the brittleness or ductility of materials based on elastic constants, and the critical value of the $B_{H}/G_{H}$ ratio is 1.75. If Pugh’s value is greater than 1.75, the material is ductile; otherwise, it is brittle. The current calculated $B_{H}/G_{H}$ ratio of CAO crystal is 2.61, showing that CAO is a ductile material. Poisson’s ratio can also be used to predict the toughness or brittleness of materials [24]. The calculated Poisson’s ratio of CAO is 0.33, which is greater than 0.26, also indicating that CAO is ductile. Manzano [25] calculated the mechanical properties of Ca_3_Al_2_O_6_, and the calculated bulk modulus, shear modulus, Young’s modulus, and Poisson’s ratio of Ca_3_Al_2_O_6_ are 102.9 GPa, 54.4 GPa, 138.7 Gpa, and 0.28, respectively. Except for Poisson’s ratio, the other parameters of mechanical behaviors of Ca_3_Al_2_O_6_ are slightly larger than those of CAO. Moreover, Ca_3_Al_2_O_6_is also a ductile material like CAO.

Elastic anisotropy of materials can be utilized to investigate some important properties such as microcracks. The universal anisotropic index ($A^{U}$) represents the elastic anisotropy of materials, and it is determined by [23]:

(9) $$A^{U}=5\frac{G_{V}}{G_{R}}+\frac{B_{V}}{B_{R}}-6>0$$

**Figure 2:**
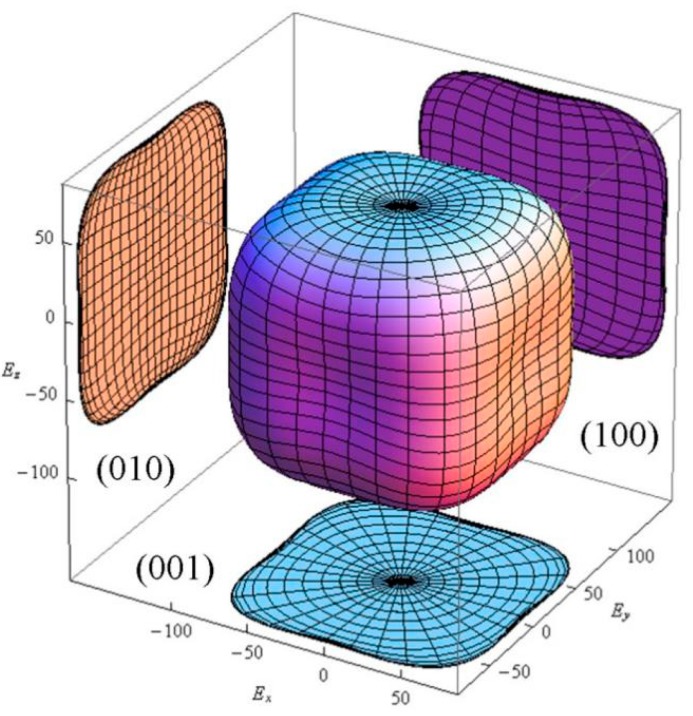
The three-dimensional Young’s modulus of CAO.

The crystal is isotropic when $A^{U}$ equals to 0. The larger the universal anisotropy index is, the greater the elastic anisotropy of the material. The calculated universal anisotropy index of CAO is 0.17, showing that CAO crystal appears to have weak anisotropy. In order to further study the elastic properties of CAO, the three-dimensional pattern of Young’s modulus for CAO crystal along different crystal faces is calculated through [26]:

(10) $$1/E=S_{11}-2\left(S_{11}-S_{12}-S_{44}/2\right)\left(l_{1}^{2}l_{2}^{2}+l_{2}^{2}l_{3}^{2}+l_{1}^{2}l_{3}^{2}\right)$$

where $S_{11}$, $S_{12}$, and $S_{44}$ are the elastic compliances, and $l_{1}$, $l_{2}$, and $l_{3}$ represent direction cosines. The three-dimensional Young’s modulus in all directions is shown in Figure 2. Figure 2 indicates the weak anisotropy of CAO, and also displays exactly the same anisotropy in the (001), (010), and (001) crystal faces.

### 3.3. Electronic Structures

**Figure 3:**
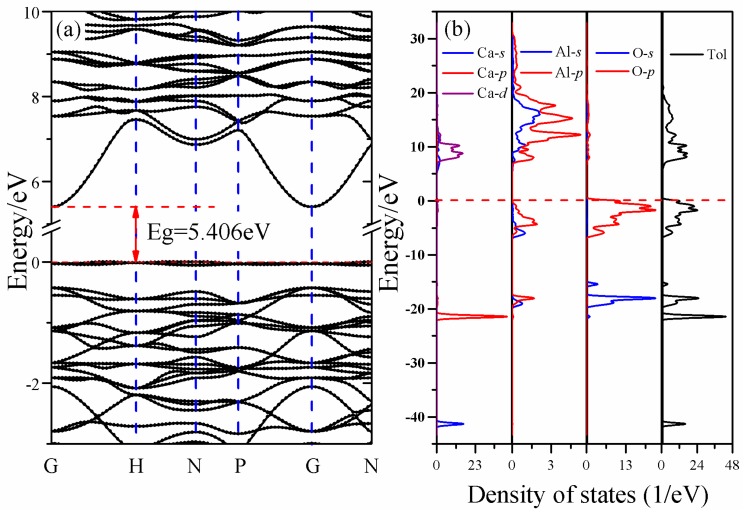
Electronic structures of CAO: (**a**) the electronic band structures and (**b**) the total and partial densities of states.

The energy band structure and total and partial densities of states of CAO are plotted in Figure 3. It can be seen that the lowest energy of the conduction band is at the high symmetry point G, while the highest energy of the valence band is at the high symmetry point H in the first Brillouin zone. This means that CAO is an indirect-gap semiconductor, and its band gap calculated by PBE0 hybrid functional is about 5.41 eV. 

However, as far as we know, there is still a lack of experimental band gap of CAO. Hussain [27] calculated the electronic structures of other common calcium aluminates, Ca_3_Al_2_O_6_, CaAl_2_O_4_, CaAl_4_O_7_, and CaAl_12_O_19_, by first principles based on the orthogonalized linear combination of atomic orbitals (OLCAO) method and found that their band gaps are 3.85, 4.16, 4.28, and 4.62 eV, respectively, which gradually increase with the increasing ratio of Al_2_O_3_. Qu et al. [28] calculated the band structures of CaAl_2_O_4_ using GGA–PBE, and the obtained band gap was 4.54 eV. The experimental band gap of CaAl_2_O_4_ is likely between 5.8 and 6.7 eV. Therefore, it can be seen that both the OLCAO and GGA–PBE methods underestimate the band gap. The band gap of CAO obtained by GGA–PBE is actually 3.51 eV. Up to now, we could deduce that the experimental band gap of CAO should be larger than 3.51 eV due to the underestimation of GGA–PBE, and smaller than 6.7 eV because of Al_2_O_3_-ratio-dependent band gaps. Simultaneously, the work of Garza et al. [20] indicates that the PBE0 hybrid functional is suitable to reproduce the band structures of solids with experimental band gaps between 3.5 and 7 eV. As a result, the current calculated band gap (5.41 eV) of CAO by the hybrid functional PBE0 is reliable. Certainly, we hope that future experimental results can directly verify our theoretical calculations.

For CAO, there are three parts in the valence bands, and the corresponding energy ranges are −42.08 to −40.47 eV, −22.28 to −14.79 eV, and −7.04 to 0.64 eV, respectively. The first part is the bottom of the valence bands (−42.08 eV to −40.47 eV) and consists of Ca 3*s* states. The second part is the lower valence bands (−22.28 eV to −14.79 eV), which is mainly made of Ca 3*p* states and O 2*s*, 2*p* states. The third part is the top valence bands (−7.04 eV to the Fermi level), a primarily composed of O 2*p* states accompanied by partial contributions of Al 3*s*, 3*p* states. The higher conduction bands (5.41 eV to 31.04 eV) mainly derive from the Ca 3*d* and Al 3*s*, 3*p* states, and O 2*s*, 2*p* and Ca 4*s* states have an ignorable influence on these bands. In the partial densities of states of CAO, the hybridization between Ca 3*d*, Al *3s*, 3*p* and O *2s*, 2*p* states can be observed. For example, the top of the valence bands are made up of hybridized Al 3*s*, 3*p* and O 2*p* states. 

### 3.4. Optical Properties

Within the linear response range, the optical response function of solids can be obtained from the optical complex dielectric function, namely:

(11) $$\varepsilon \left(\omega \right)=\varepsilon_{1}\left(\omega \right)+i\varepsilon_{2}\left(\omega \right)$$

The imaginary part $\varepsilon_{2}\left(\omega \right)$ describes energy losses encountered in polarizing solids and reflects electron transitions from the occupied states to the unoccupied states [29], while the real part $\varepsilon_{1}\left(\omega \right)$ represents the polarization strength induced by an external electric field and can be obtained from the imaginary part $\varepsilon_{2}\left(\omega \right)$ by using the Kramers–Kroing dispersion relation [30].

Other optical properties, such as the refractive index (n), extinction coefficient (k), absorption coefficient (I), reflectivity index (R), energy loss function (L), and conductivity (σ), can be obtained through the real and imaginary parts of the dielectric function as follows [29]:

(12) $$n\left(\omega \right)=\frac{{\left[\sqrt{\varepsilon_{1}^{2}\left(\omega \right)+\varepsilon_{2}^{2}\left(\omega \right)}+\varepsilon_{1}\left(\omega \right)\right]}^{\frac{1}{2}}}{\sqrt{2}}$$

(13) $$k\left(\omega \right)=\frac{{\left[\sqrt{\varepsilon_{1}^{2}\left(\omega \right)+\varepsilon_{2}^{2}\left(\omega \right)}-\varepsilon_{1}\left(\omega \right)\right]}^{\frac{1}{2}}}{\sqrt{2}}$$

(14) $$I\left(\omega \right)=\sqrt{2}\omega {\left[\sqrt{\varepsilon_{1}^{2}\left(\omega \right)+\varepsilon_{2}^{2}\left(\omega \right)}-\varepsilon_{1}\left(\omega \right)\right]}^{\frac{1}{2}}$$

(15) $$R\left(\omega \right)=\frac{{\left(n\left(\omega \right)-1\right)}^{2}+k^{2}\left(\omega \right)}{{\left(n\left(\omega \right)+1\right)}^{2}+k^{2}\left(\omega \right)}$$

(16) $$L\left(\omega \right)=\frac{\varepsilon_{2}\left(\omega \right)}{\varepsilon_{1}{}^{2}\left(\omega \right)+\varepsilon_{2}{}^{2}\left(\omega \right)}$$

(17) $$\sigma \left(\omega \right)=\frac{\omega \varepsilon_{2}\left(\omega \right)}{4\pi }$$

**Figure 4:**
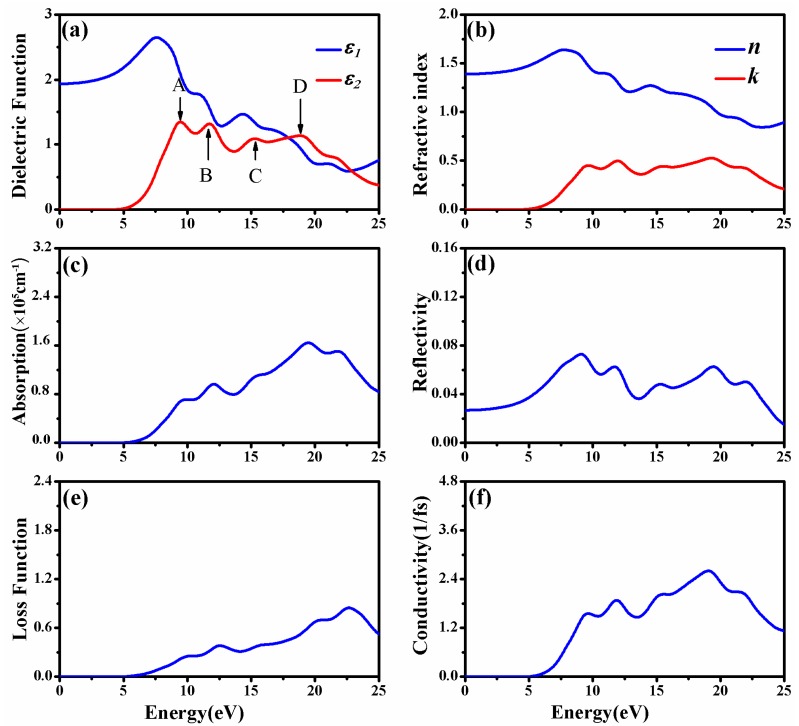
Optical properties of CAO: (**a**) Dielectric function; (**b**) refractive index, extinction coefficient; (**c**) absorption coefficient; (**d**) reflectivity; (**e**) loss function; and (**f**) conductivity.

In order to depict as many of the details of electronic transitions in CAO as possible, the number of chosen empty bands during calculations was three times the number of valence bands. At this time, the dielectric functions were calculated up to 75 eV; however, only the meaningful energy region less than 25 eV was analyzed. Figure 4a shows the real and imaginary parts of dielectric function of CAO. The value of $\varepsilon_{1}\left(\omega \right)$ is always larger than 0 in the energy range from 0 to 25 eV, indicating that incident light can always propagate in CAO in this energy range. The static dielectric constant $\varepsilon_{1}\left(0\right)$ equals 1.94. The imaginary part of the dielectric function has four obvious peaks at 9.45, 11.74, 15.29 and 18.89 eV, named A, B, C, and D, respectively, in Figure 4a. According to the energy band structure and the density of states in Figure 3, peak A represents the valence electron transitions from O 2*p* states of valence bands to Ca 3*d* and Ca4*s* states; peak B corresponds to transitions from O 2*p* states to empty Ca 3*d*, 4*s* states; peak C emerges from the transitions from O 2*p* to Al 3*s*, 3*p* conduction bands; and peak D represents the transitions from O 2*p* valence bands to Al 3*s*, 3*p* states.

Figure 4b displays the refractive index n and extinction coefficient k of CAO. The changing trend of the refractive index is similar to that of the real part of the dielectric function. From the refractive index, the static refractive index of CAO is 1.39, and the maximum refractive index corresponds to the photon energy of 7.74 eV. The extinction coefficient is zero in the visible region, meaning that CAO has favorable transmittance within this energy region. In the ultraviolet zone, there are five peaks of the extinction coefficient located at 9.71, 11.96, 15.38, 19.32, and 21.77 eV, respectively. The positions of these peaks correspond to the peaks in the imaginary part of the dielectric function (see Figure 4b).

Hussain [27] calculated the frequency-dependent complex dielectric functions and estimated the refractive indices of common calcium aluminates, including Ca_3_Al_2_O_6_, Ca_12_Al_14_O_33_, CaAl_2_O_4_, CaAl_4_O_7_, and CaAl_12_O_19_. Their corresponding static dielectric constants $\varepsilon_{1}\left(0\right)$ are 3.417, 2.766, 3.034, 2.759, and 3.253, respectively. The static dielectric constant and refractive index of CAO are slightly smaller than those of other common calcium aluminates.

Figure 4c shows the absorption coefficient of CAO. The calculated absorption edge is 5.36 eV and close to the band gap, indicating that CAO begins to absorb radiation when the wavelength is below 187.9 nm, located at the ultraviolet region. The reflectivity of CAO shown in Figure 4d indicates that this crystal reflects hardly any light, because the maximum reflectivity is about 9% in the range of 0 to 25 eV and only 3% in the visible region. The high absorption coefficient and low reflectivity show that CAO can be used as a UV-absorbent material in photoelectric devices.

Figure 4e is the loss function of CAO. The loss function is used to describe the energy loss when the electron passes through materials. The oscillation frequency, corresponding to the peak in the loss function, is the bulk plasma frequency [31]. As for CAO, photoelectron energy loss occurs in energy regions larger than 5 eV. Figure 4f reflects the relationship between the conductivity of CAO and the photon energy. Photoconductivity describes the phenomenon where by the incident light causes a change in the conductivity of a semiconductor. It is an important parameter of optoelectronic materials, and is closely related to the photoelectric conversion efficiency. The conductivity of CAO is 0 in the visible range, and higher in the energy ranges of 8.70 to 25 eV.

### 3.5. Phonon Spectra

**Figure 5:**
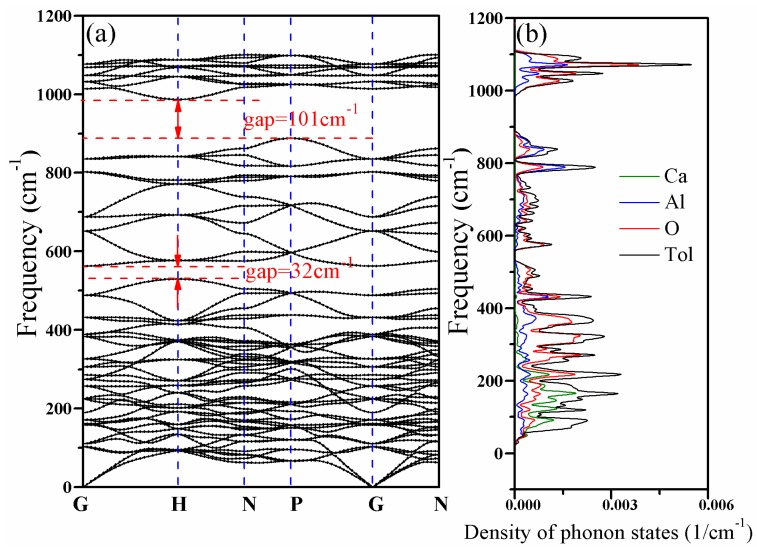
Phonon dispersion relations and density of phonon states of CAO. (**a**) Phonon dispersion relations and (**b**) density of phonon states.

Figure 5 shows the phonon dispersion relations and the density of phonon states of CAO. There are 23 atoms in the primitive cell of CAO. Accordingly, this crystal has 69 kinds of dispersion relations, including three acoustic branches and 66 optical branches. The acoustic branches reflect vibrations of the cell centroid, while the optical branches represent the relative vibrations between atoms. We can see that the phonon dispersions have no imaginary frequency in the whole Brillouin zone, proving that CAO is dynamically stable at ground state for the given structure. 

To further comprehend the behaviors of the optical phonon branches, lattice vibrations should be discussed in more depth. CAO belongs to the *I*-43 m space group, whose point group is *T*_d_. Based on the standard group theory analysis, the irreducible representation of optical branches of vibration modes at point G in the first Brillouin zone is given by:

(18) $$G_{\mathrm{opt}}=10T_{2}^{\left(R+\mathrm{IR}\right)}+7T_{1}^{\left(S\right)}+5E^{\left(R\right)}+2A_{2}^{\left(S\right)}+3A_{1}^{\left(R\right)}$$

In Equation (18), the E and A_1_ modes are Raman active (R), the T_2_ mode is both Raman and infrared active (IR), and the T_1_ and A_2_modes are silent modes (S). There are, in total, 30 T_2_ modes, 21 T_1_ modes, 10 E modes, two A_2_ modes, and three A_1_ modes, and they collectively consist of 66 optical dispersion relations. Each T mode is three-fold degenerate, E mode is double-degenerate, and A mode is non-degenerate [32]. The characteristics of lattice vibrational spectra depend on the chemical bonding strengths and masses of structural units [33]. At the high-symmetric point G, all phonon modes are double degenerate or three fold degenerate, while all branches are non-degenerate at point N. The phonon dispersion curves of CAO have two band gaps with widths of 101.3 cm^−1^ and 32 cm^−1^, because of the large difference of atomic mass between Ca, Al, and O atoms. From the phonon density of states of CAO, the maximum peak appears at a frequency of 1067.1 cm^−1^, indicating that the lattice vibration is most violent in the vicinity of 1067.1 cm^−1^. Furthermore, lattice vibrations primarily occur in the frequency ranges of 23 to 532 cm^−1^, 555.7 to 892.9 cm^−1^, and 980.7 to 1119.3 cm^−1^. In the relatively high-frequency region (980.7 to 1119.3 cm^−1^) and the frequency ranges of 555.7 to 892.9 cm^−1^ and 241.9 to 532 cm^−1^, the vibrations of Al atoms and O atoms contribute dominantly to the lattice vibrations. In the low-frequency area (23 to 241.9 cm^−1^), the lattice vibrations are mainly caused by the vibration of Ca atoms and partly come from the vibrations of Al atoms and O atoms. 

**Figure 6:**
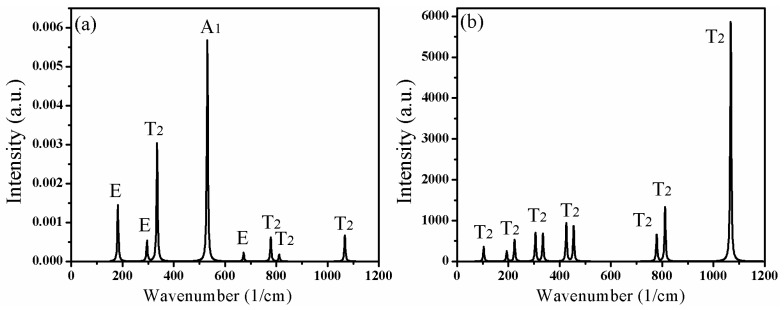
Lattice vibrations and the corresponding active modes of CAO. (**a**) Raman scattering spectra and (**b**) IR absorption spectra.

Table 2 lists the calculated optical phonon frequencies (cm^−1^) and the corresponding vibration modes in the Brillouin zone center. The vibrations in Raman scattering spectra include T_2_, E, and A_1_ modes, while the infrared active vibrations contain only T_2_ modes. Figure 6 further illustrates the intensity of vibration modes corresponding to different frequencies in the Raman scattering and infrared absorption spectra of CAO. Figure 6a shows that the highest peak is the A_1_ mode at 270 cm^−1^, and the second one is the T_2_ mode at 306 cm^−1^. The above two modes only involve the vibrations of O atoms. Moreover, the A_1_ mode at 565 cm^−1^ also has relatively high intensity, which derives from the vibrations of O atoms. From Figure 6b, the strongest peak is at 1046 cm^−1^, corresponding to the T_2_ mode, which comes from the vibrations of Al and O atoms.

**Figure 7:**
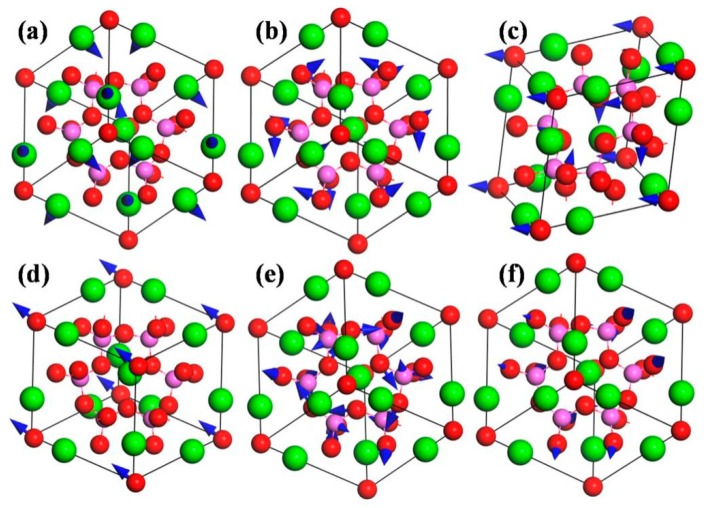
Atomic displacement patterns of CAO. (**a**) E 180 cm^−1^; (**b**) A_1_ 270 cm^−1^; (**c**) T_2_ 306 cm^−1^; (**d**) T_2_ 337 cm^−1^; (**e**) A_2_ 1012 cm^−1^; and(**f**) T_2_ 1046 cm^−1.^

In order to understand the vibration modes more intuitively, the typical atomic displacement patterns of CAO are displayed in Figure 7. The directions of atomic displacements can be observed from the primitive cell of CAO in the 2 × 2 × 2 super cell (see Figure 1b). Figure 7a is the E mode at 180 cm^−1^, which involves the vibrations of all Ca atoms in the primitive cell of CAO, while Al and O atoms remain stationary. The vibration directions of Ca atoms on the edges are [$\bar{1}$21], [$\bar{1}$1$\bar{2}$], and [1$\bar{1}\bar{2}$], respectively, and the central Ca atom vibrates along the [$\bar{1}$12] direction. From Figure 7b, the atomic displacement mode at 270 cm^−1^ corresponds to the A_1_mode, and mainly involves the vibrations of Al atoms. The vibration directions of Al atoms are [1$\bar{1}\bar{1}$], [11$\bar{1}$], [$\bar{1}\bar{1}$1], and [111], respectively, resulting in swinging of Al–O bonds. Figure 7c is the vibrations of T_2_ mode at 306 cm^−1^, which are only composed of the vibrations of O atoms. O atoms at the vertex angle vibrate along [1$\bar{2}$1], and the main vibration directions of the remaining O atoms are [111] and [$\bar{1}\bar{1}\bar{1}$]. Figure 7d is T_2_ mode at 337 cm^−1^, which involves only the same-direction vibrations of O atoms at apex angle, and their vibration direction is [$\bar{1}$12]. The A_2_ silent mode at 1012 cm^−1^ (see Figure 7e) involves the vibrations of O and Al atoms, while Ca atoms keep still. O atoms vibrate in the directions of <101>, and the vibration directions of Al atoms are <100>. The vibrations of O and Al atoms can give rise to stretching and torsion motions of Al–O bonds. Figure 7f is the T_2_ mode at 1046 cm^−1^, which mainly comes from the vibrations of Al and O atoms. The vibration directions of O atoms are [0$\bar{1}$1], [1$\bar{1}$0], and [$\bar{1}$01], and Al atoms in the Al–O tetrahedron have very small vibrations, leading to stretching and torsion of Al–O bonds with different amplitudes.

## 4. Conclusions

The electronic structures, mechanical behaviors, and optical properties of CAO crystal were studied in this work. Similar to sodalite, CAO belongs to the cubic system. The sodalite cage is comprised of 24 skeleton atoms, and they form eight hexatomic rings and six tetra-atomic rings. CAO is a weakly anisotropic ductile material. The electronic band structures confirm that CAO crystal is also an indirect-gap semiconductor, and the band gap is 5.41 eV. The valence band top is made up of O 2*p* states, and the bottom conduction band is composed of Ca 3*d* states. The transitions from O 2*s*, 2*p* states to empty Ca 4*s*, 3*d* and Al 3*s*, 3*p* states constitute the peaks of the imaginary part of the dielectric function in CAO. Moreover, CAO can be used as a UV absorber material in photoelectric devices, due to its high absorption coefficient and low reflectivity in the ultraviolet zone. The lattice vibration analysis of CAO reveals that O atoms contribute to the high-frequency portions of the phonon spectra, while the Ca and Al atoms make important contributions to the middle- and low-frequency portions. There are 69 dispersion relations in total, and the optical branches are made up of 30 T_2_ modes, 21 T_1_ modes, 10 E modes, two A_2_ modes, and three A_1_ modes. The Raman active vibrational modes include T_2_, E, and A_1_ modes, and the A_1_ mode at 270 cm^−1^ involved only the vibrations of O atoms, which have the highest intensity. The infrared absorption spectra contain only T_2_ modes, and the highest peak is located at 1046 cm^−1^, which derives from vibrations of Al and O atoms. The different atomic displacement patterns display the direction and amplitude of the vibrations of Ca, Al, and O atoms at the corresponding frequency.

## Figures and Tables

**Table 1:** The calculated bulk moduli ($B_{V}$, $B_{R}$ and $B_{H}$, in GPa), shear moduli ($G_{V}$, $G_{R}$ and $G_{H}$, in GPa), Young’s moduli (E, in GPa), and Poisson’s ratio (ν) of CAO.

$B_{V}$	$B_{R}$	$B_{H}$	$G_{V}$	$G_{R}$	$G_{H}$	E	ν
93.18	93.18	93.18	36.25	35.05	35.65	77.53	0.33

**Table 2:** The calculated optical phonon frequencies (cm^−1^) of vibration modes and the atoms involved in the vibrations at point G in the first Brillouin zone.

Modes	Frequencies and Atoms Involved				
T_2_^(R+IR)^	132 (Ca, Al, O)	197 (Ca, Al, O)	246 (Ca, O)	306 (O)	337 (O)
	385 (Al, O)	428 (Al, O)	801 (Al, O)	835 (Al, O)	1046 (Al, O)
T_1_^(S)^	108 (Ca, Al, O)	177 (Ca, Al, O)	263 (Al, O)	385 (Al, O)	651(Al, O)
	1089 (Al, O)	1075 (Al, O)	-	-	-
E^(R)^	180 (Ca)	262 (Al, O)	492 (Al, O)	686 (Al, O)	1030 (Al, O)
A_2_^(S)^	183 (Al, O)	1012 (Al, O)	-	-	-
A_1_^(R)^	270 (Ca, O)	284 (Ca, O)	565 (O)	-	-
